# The Influence of Density on the Value of Young’s Modulus for Dry Ice

**DOI:** 10.3390/ma14247763

**Published:** 2021-12-15

**Authors:** Aleksandra Biszczanik, Krzysztof Wałęsa, Mateusz Kukla, Jan Górecki

**Affiliations:** Faculty of Mechanical Engineering, Institute of Machine Design, Poznan University of Technology, 60-965 Poznań, Poland; aleksandra.biszczanik@put.poznan.pl (A.B.); krzysztof.walesa@put.poznan.pl (K.W.); mateusz.kukla@put.poznan.pl (M.K.)

**Keywords:** Young’s modulus, compaction, dry ice, energy consumption, working load estimation

## Abstract

The efficiency of material consumption is an important consideration for production processes; this is particularly true for processes that use waste materials. Dry ice extrusion serves as a good example. An examination of the literature on this subject leads to an observation that the commercially available machines for dry ice compression are characterized by a high value of working force. Consequently, the effectiveness of the source consumption, electric energy and carbon dioxide, is very low. The subject of the experimental research presented in the article is the influence of the density of dry ice on the value of Young’s modulus. The first part of the article presents the test methodology and the special test stand that was developed to accommodate the unique characteristics of solid-state carbon dioxide. The test results present the characteristics of compaction and relaxation used as the basis for determining the value of Young’s modulus. Based on the test results obtained for various material density values, the characteristics of Young’s modulus are developed and graphed as a function of the density. The presented results are important for furthering the research on the development of extrusion and compaction processes; for example, using the Drucker–Prager/Cap model for the purpose of optimizing the geometrical characteristics of the work assembly components.

## 1. Introduction

For many years, manufacturing facilities have made efforts to improve the use of waste material. Apart from ensuring the correct storage of such materials, they are frequently reintroduced into the manufacturing process, or the waste material is processed to be used in other manufacturing activities.

**Figure 1:**
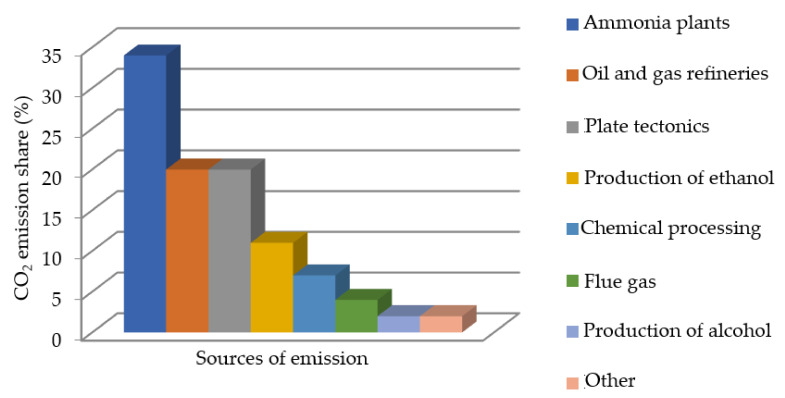
Main sources of carbon dioxide emissions by percentage share [7].

An example of such a waste material is carbon dioxide, which is generated in high quantities during the manufacturing of ammonia compounds [1,2], as illustrated in Figure 1. Whilst on the premises of the manufacturing facility, the carbon dioxide is compressed in its gaseous state until a pressure of approximately 20 bar is achieved, causing liquefaction [1]. In its liquid form, the material can be used to manufacture dry ice via rapid decompression to atmospheric pressure. The resultant material exhibits unique characteristics; for example, a temperature value of −78.4 °C, as well as sublimation in atmospheric conditions [3,4,5,6].

The dry ice in its fragmented form, obtained after decompression, does not have a wide range of applications. This is due to its intensive sublimation under normal conditions. To reduce the intensity of this phenomenon, the material is compacted and extruded, typically in the form of a cylindrical pellet, with a diameter between 3 and 16 mm. In this form, the material is used for refrigeration [6,7,8,9], as well as for surface preparation and cleaning [10,11,12,13,14,15,16,17,18,19]. Due to the growing number of possible applications, the energy consumption of the compaction process becomes a significant consideration, which is related to the optimization of the working load value of the relevant machines.

The issue of the efficiency of the compaction process applies to many different types of waste material. Consequently, we have observed an increasing number of studies that focus on the modeling of industrial processes for the purpose of estimating the working load [20,21,22,23]. Wilczyński presented the results of a study that led to the development of a compaction process for materials intended to be used in the manufacturing of biofuels [24]. The publication presented the results of the laboratory research and a numerical analysis performed in relation to the compaction process. For the purpose of process simulation, the Drucker–Prager/Cap (DPC) material plasticity model was used. The use of numerical simulations allowed the determination of the values of effective process parameters. Berdychowski further indicated the possibility of employing two material plasticity models other than the DPC [25], i.e., Mohr–Coulomb (MC) [26] and Cam–Clay (CC) [27].

The DPC, MC and CC models are relevant for the process, with regards to simulation of the plastic deformation of the compacted material. To fully represent the process, the simulations were supplemented with a model to describe the elastic strain. To this end, a linear model of elasticity was employed, which required an input value to represent the modulus of the material elasticity, referred to as the Young’s modulus *E* [28].

Sufficient information on the mechanical parameters of the material was not found in the available subject literature. Therefore, to begin the research and development work, to improve the energy efficiency of the CO_2_ compaction process, it was necessary to perform the examinations presented herein.

## 2. Materials and Methods

The studies available in subject literature present methodologies to determine the sought characteristic curve of Young’s modulus variance as a function of material density [24,29]. The values of Young’s modulus for specific material density values are determined based on the characteristic curves of compression and relaxation. The subject literature assumes that during material relaxation, its strain characteristics are elastic. Therefore, the Young’s modulus *E* is equal to the absolute value of the gradient of the stress value change *σ* as a function of the absolute strain *ε* at the section in which the material is unstressed. The indicated dependence can be expressed by the following formula:

(1) $$E=\left|\nabla \sigma \left(\varepsilon \right)\right|=\left|\frac{d\sigma \left(\varepsilon \right)}{d\varepsilon }\right|$$

If the resulting correlation value is high at the material section of relaxation, the value *E* may be determined by the use of linear regression using the least square method.

The applied study methodologies and used testing stations were not adapted to the peculiar characteristics of dry ice under normal conditions, i.e., a temperature value of 78.4 °C and sublimation. Therefore, this article presents a designed testing station together with a developed methodology, which were adapted to the characteristics of dry ice.

For the purpose of this study, dry ice in a loose form with a bulk density of approximately 500 kg/m^3^ was used, which—after compression—can achieve a maximum density of approximately 1650 kg/m^3^. The material was kept in an insulated container made of foamed polystyrene to limit the intensity of sublimation resulting from heat being supplied from the surroundings, as well as to limit the possibility of condensation and the crystallization of steam in the ambient air.

The aim of the examination was to determine the elastic modulus *E* of dry ice as a function of the material density *ρ*. The employed method was used to determine the curves for compression with relaxation for an assigned material density. To this end, the dry ice compaction was performed in a specially designed station until the specific material density was achieved. Afterwards, the compressing force was withdrawn and the system was allowed to move freely. Over the course of the examination, the force value applied by the piston and its displacement were recorded.

**Figure 2:**
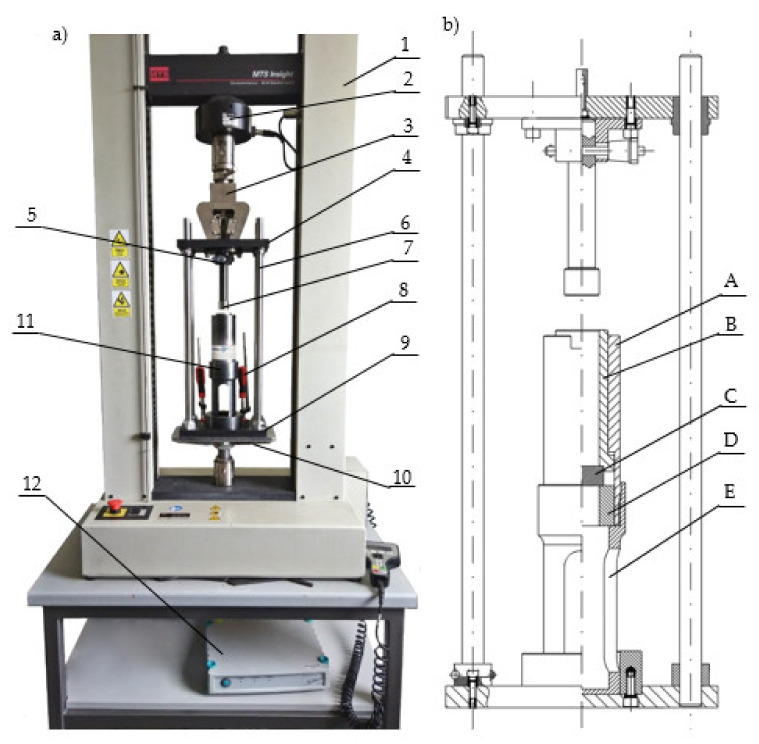
Testing station for the examination of the elastic modulus of dry ice as a function of the density. (**a**) 1: MTS durometer; 2: machine sensor; 3: machine grip; 4: upper plate; 5: Kipp locking pin; 6: guide assembly; 7: piston; 8: clamps; 9: lower plate; 10: machine base; 11: compacting sleeve assembly; 12: Spider 8 instrumentation amplifier. (**b**) Compacting sleeve assembly, cross-section view. A: upper sleeve; B: compacting chamber; C: compacting chamber bottom; D: spacer sleeve; E: lower sleeve.

The study used an MTS Insight 50 kN durometer equipped with a tensometric sensor for measuring the force value and a displacement sensor with an accuracy class of 0.5. A measurement station for the empirical verification of the compacting stress value during the agglomeration of the dry ice was installed between the machine grips. The device setup, as shown in Figure 2a, facilitated successful examinations and enabled the value of the force applied to the compressing piston to be recorded as a function of its displacement. The signals received from the sensors were routed to an HBM Spider 8 instrumentation amplifier and were subsequently received by Catman Easy software by the HBM. The process of the acquisition and processing of both measurement signal values was performed at a frequency of 100 Hz. The measurements of the mass of the loose input material and the final compacted product sample were identified using a KERN and SohnGmbH laboratory scale, model PCB10000-1, with measurement accuracy of up to 0.1 g.

Figure 2b provides a more detailed view of the testing station for the compaction and relaxation of agglomerated dry ice, as well as the internal construction of the compacting sleeve assembly.

Before starting the tests, the air temperature in the room was lowered to the value of 18 °C and the value of air humidity did not exceed 50%.

The testing station was first placed on the durometer to perform the initial setup to ensure the concentric arrangement of its components. This process entailed arranging the assembled testing station so that the piston (7, Figure 2a) was installed on the upper plate (4, Figure 2a) with the locking pin (5, Figure 2a) placed inside the compacting chamber (B, Figure 2b) during the installation of the main plate (4, Figure 2a) in the durometer grip (3, Figure 2a). The clamps (8, Figure 2a) were used in a sequence to attach the lower plate of the testing station (9, Figure 2a) and the guide assembly (6, Figure 2a) to the plate built into the durometer (10, Figure 2a), for the purpose of immobilizing the assembly. Furthermore, it was tested to ensure no additional resistance occurred during motion. After the setup, the compacting sleeve assembly (11, Figure 2a) and the piston (7, Figure 2a) were removed from the station and placed in the dry ice container to cool down for approximately 45 min. The compacting sleeve assembly and piston were cooled down with dry ice to reduce their temperature, achieving a temperature value similar to the tested material, −65 °C. Temperature of the chamber was measured using thermocouple sensor type K. After this, the testing station was reassembled and the piston surface was placed in the reference position in relation to the compacting chamber, and the testing station was cooled down for another 5 min. The compacting sleeve assembly was then placed on the scale to measure the weight of the assembly; the determined amount of dry ice, depending on the expected test sample density after compression, was then placed inside the compacting chamber with a diameter of 29.95 mm, measured after cooling. Subsequently, the piston together with the compacting sleeve assembly were mounted in the guide assembly, fixed in the durometer grips and the measurement was initiated according to the algorithm programmed into the TestWorks 4 software that controlled the MTS machine. Simultaneously, the result acquisition was initiated with Catman Easy software. The machine performed the following programmed sequence:

1. The upper grip of the durometer together with the piston arrived at the reference position with the initial velocity.
2. An initial downward motion was instigated with a travel speed of 1 mm/s until a resistance force value of 150 N was detected.
3. The test was initialized. The machine grip moved downwards together with the installed upper plate and the piston at a test speed of 5 mm/s until the sample height value of 24 ± 0.05 mm was achieved.
4. The assembly retracted with a speed of 5 mm/s until a force value of 0 N was achieved.
5. The piston retracted with the end of the test speed to a height approximately 60 mm above the reference position to facilitate the removal of the sleeve and sample for weighing.

After the test sample was completed, the sleeve assembly was weighed once more to determine the mass of the compacted dry ice sample and minimize the loss of mass due to sublimation as much as possible; the actual sample was then removed from the assembly and weighed (the difference in the measurement was approximately 0.1 g, similar to the measurement accuracy of the used scales). The testing station components were then placed in dry ice for 5 min to cool prior to performing the next test.

The examination was performed for six levels of material compaction. To achieve the desired material density, the following amounts were successively placed in the chamber: 20 g (final density, approximately 1050 kg/m^3^); 22 g (final density, approximately 1150 kg/m^3^); 24 g (final density, approximately 1250 kg/m^3^); 26 g (final density, approximately 1320 kg/m^3^); 28 g (final density, approximately 1410 kg/m^3^); 30 g (final density, approximately 1590 kg/m^3^). A total of 10 tests were performed for each density value. Based on the statistical analysis of the results, three samples for each density were discarded with the most divergent values from the recorded average during the examination.

## 3. Results

**Figure 3:**
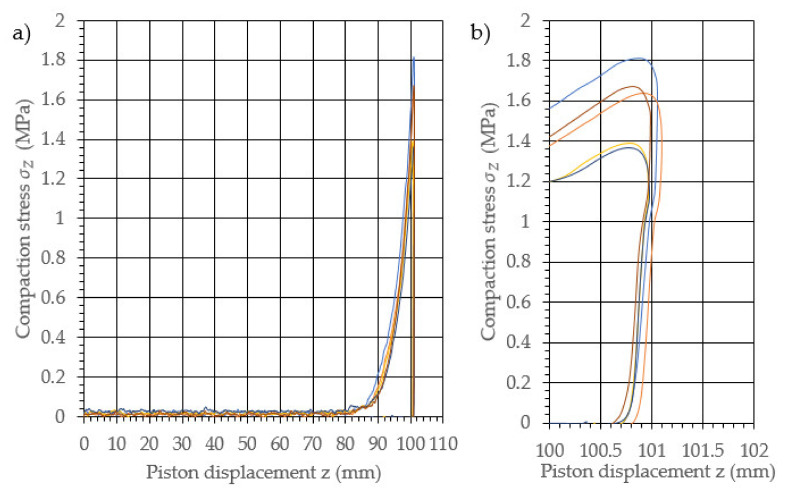
Characteristic curves for the compression with relaxation of dry ice with a final density of 1065.0 kg/m^3^. (**a**): The overall course of the characteristics; (**b**): an enlarged relaxation section of the compacted material.

**Figure 4:**
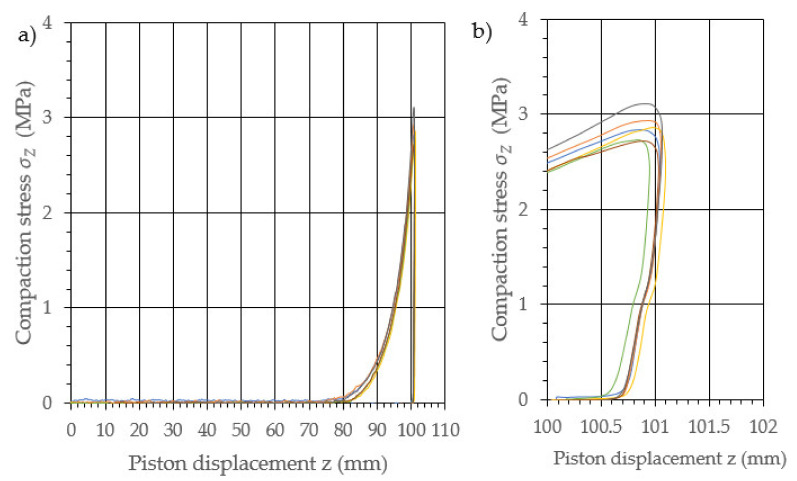
Characteristic curves for the compression with relaxation of dry ice with a final density of 1164.9 kg/m^3^. (**a**): The overall course of the characteristics; (**b**): an enlarged relaxation section of the compacted material.

**Figure 5:**
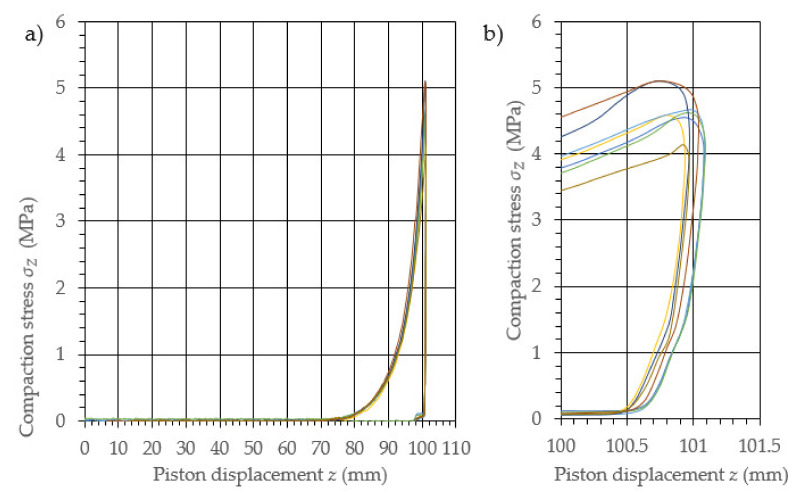
Characteristic curves for the compression with relaxation of dry ice with a final density of 1225.2 kg/m^3^. (**a**): The overall course of the characteristics; (**b**): an enlarged relaxation section of the compacted material.

**Figure 6:**
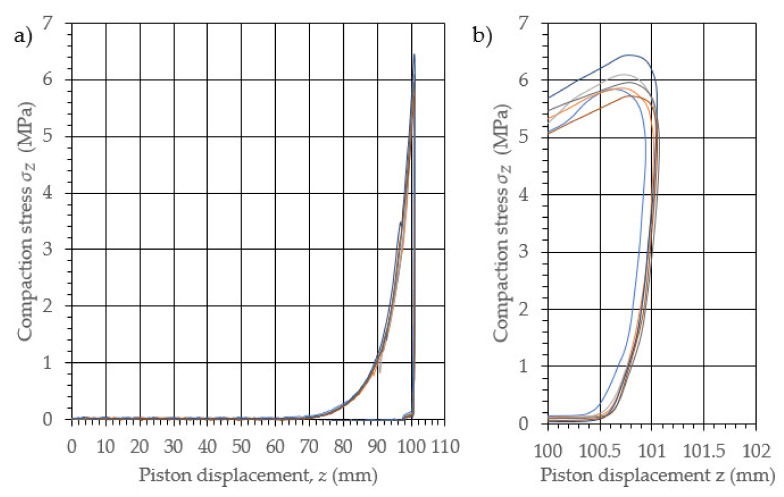
Characteristic curves for the compression with relaxation of dry ice with a final density of 1321.3 kg/m^3^. (**a**): The overall course of the characteristics; (**b**): an enlarged relaxation section of the compacted material.

**Figure 7:**
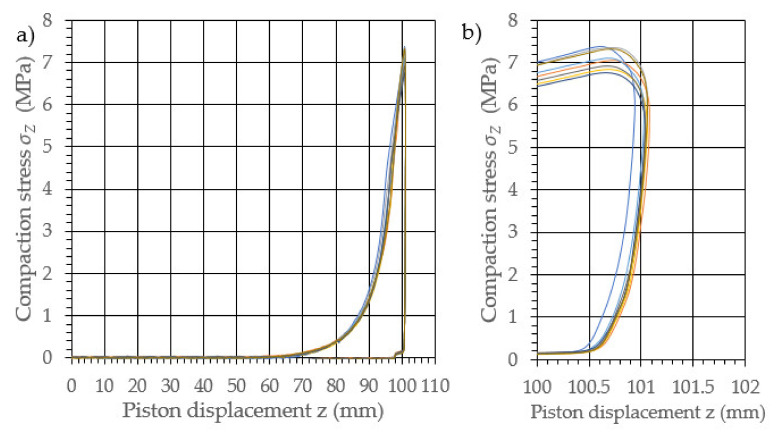
Characteristic curves for the compression with relaxation of dry ice with a final density of 1412.3 kg/m^3^. (**a**): The overall course of the characteristics; (**b**): an enlarged relaxation section of the compacted material.

**Figure 8:**
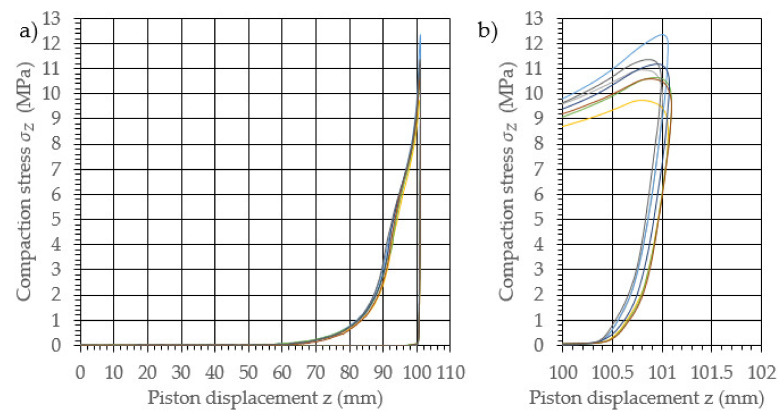
Characteristic curves for the compression and relaxation of dry ice with a final density of 1574.5 kg/m^3^. (**a**): The overall course of the characteristics; (**b**): an enlarged relaxation section of the compacted material.

The performed study allowed the development of the characteristics of the compaction with relaxation for each examined density of dry ice agglomerate. The results are illustrated in Figure 3, Figure 4, Figure 5, Figure 6, Figure 7 and Figure 8. The measured results were characterized by a high degree of conformity, with the standard deviation of the compacting force threshold values not exceeding 11.0% of the average value.

Due to dry ice sublimation over the course of the test, the density value was determined at the end of each test. Table 1 provides the averaged results of the measurements of the initial sample weight *m*_0_, the final sample weight *m*_1_, the sample height *h*, the sample diameter *d*, and the density *ρ* for each type of sample. For the parameters *m*_1_, *h*, and *ρ*, standard deviation values, *σ*, are provided.

**Figure 9:**
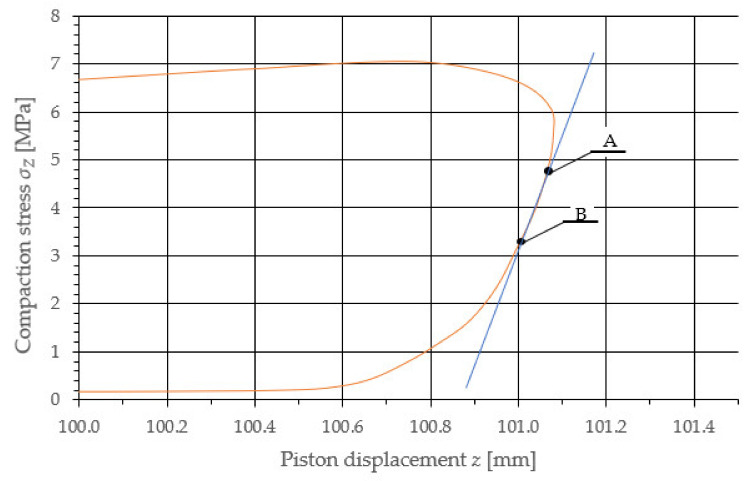
Approximate section describing the variance in the compressing stress value during the sample relaxation indicating the starting point A and the end point B of the linear regression for the selected sample characteristic with a density equal to 1412.3 kg/m^3^.

To determine the value of Young’s modulus, the parameter value was determined for the material release section for each of the curves. The example points of the measurements used to determine the value of the sought material parameters are indicated in Figure 9.

For the purpose of determining the value of Young’s modulus, a function describing the change in the compacting stress value between points A and B was established, by means of REGLINP software for linear regression using the least squares method, available in Microsoft Excel. For the determined function, the directional parameter value was equal to the value of Young’s modulus. The calculations provided herein were performed for each of the determined characteristics, and the average values of each of the examined sample densities are provided in Table 2.

**Figure 10:**
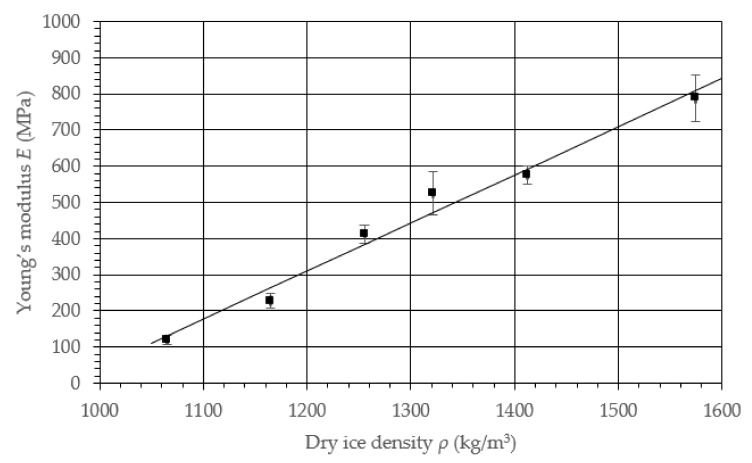
Characteristic curve for the Young’s modulus *E* value change as a function of the density *ρ* for dry ice.

The results provided in Table 2 are presented in the graph in Figure 10. The result correlation was equal to 0.9569; therefore, the path of the characteristic curve could be described with the following function:

(2) $$E\left(\rho \right)=1.328\rho -1282.93$$

The presented equation could be applied to a limited range of density values. This followed on from the limit value of the material density equal to 1625 kg/m^3^ [30], as well as the lack of cohesion of the materials with a density below 1000 kg/m^3^.

## 4. Conclusions and Results Discussion

The provided study results allowed the determination of the characteristic curve for the change in the value of Young’s modulus during dry ice compaction.

On the basis of the determined equation, it is possible to determine the value of Young’s modulus at each stage of the dry ice compaction process. This equation can be used in numerical studies concerning the description of the processes of compaction (using, for example, DPC, CC and MC models [25,26,28,31,32]), and extrusion (with the use of the elastic-plastic model [33]) of dry ice.

The illustrated characteristics of compression and relaxation are, therefore, to be considered a supplementation of the characteristics describing the section of relaxation available in the subject literature [28]. The progressive characteristic curve supported the conclusion formulated in the subject literature, which was that dry ice agglomerates should be qualified as a brittle material [30]. The previously indicated DPC, MC and CC models were used when simulating the densification process of brittle materials [31,34,35], which provided examination results that were only applicable within a specific range of material density values. This follows on from the maximum density value for this material, equal to 1625 kg/m^3^ [30], and the lack of cohesion of materials with a density below 1000 kg/m^3^.

The results of the study could be applied to further research involving the following:

- Numerical simulation of the compaction and extrusion processes using the DPC, CC and MC material models, for the purpose of estimating the working load;
- Optimization of the geometric characteristics of the tools used in the processes of compaction and extrusion of dry ice, to increase the process efficiency;
- Analysis of the energy consumption of the dry ice palletization process with the use of a gravity roller press.

## Figures and Tables

**Table 1:** Sample density measurement results.

*m*_0_ (g)	$m_{1}\;(\pm \sigma_{A}^{m_{1}})\;(g)$	*d* (mm)	$h\;(\pm \sigma_{A}^{h})\;(\mathrm{mm})$	$\rho \;(\pm \sigma_{A}^{\rho })\;(\mathrm{kg}/m^{3})$
20	18.06 (0.32)	30	23.99 (0.04)	1065.0 (20.69)
22	19.73 (0.22)	30	23.96 (0.04)	1164.9 (12.78)
24	21.27 (0.29)	30	23.98 (0.06)	1255.2 (17.79)
26	22.39 (0.55)	30	23.96 (0.04)	1321.3 (33.26)
28	23.94 (0.52)	30	23.98 (0.05)	1412.3 (28.97)
30	26.66 (0.36)	30	23.95 (0.03)	1574.5 (20.66)

**Table 2:** Young’s modulus values for selected dry ice sample densities.

*ρ* (kg/m^3^)	$\sigma_{A}^{\rho }$ (kg/m^3^)	E (MPa)	$\sigma_{A}^{E}$ (MPa)
1065.0	20.69	120.82	12.9
1164.9	12.78	228.18	20.47
1255.2	17.79	411.84	24.23
1321.3	33.26	525.03	60.07
1412.3	28.97	576.73	25.55
1574.5	20.66	788.13	63.09

## Data Availability

Not applicable.
